# Robust Synthesis of Size-Dispersal Triangular Silver Nanoprisms via Chemical Reduction Route and Their Cytotoxicity

**DOI:** 10.3390/nano9050674

**Published:** 2019-05-01

**Authors:** Hagar S. Bahlol, Mohamed F. Foda, Jing Ma, Heyou Han

**Affiliations:** 1 State Key Laboratory of Agricultural Microbiology, College of Science, Huazhong Agricultural University, Wuhan 430070, China; hagar.shendy@webmail.hzau.edu.cn (H.S.B.); m.frahat@fagr.bu.edu.eg (M.F.F.); majing@yangtzeu.edu.cn (J.M.); 2 Department of Biochemistry, Faculty of Agriculture, Benha University, Moshtohor, Toukh 13736, Egypt; 3 State Key Laboratory of Agricultural Microbiology, College of Veterinary Medicine, Huazhong Agricultural University, Wuhan 430070, China

**Keywords:** direct chemical reduction, silver nanoparticles, nanoprisms, silica coated nanoprisms, cell viability assay

## Abstract

Triangular silver nanocrystals, well-known as nanoprisms (Ag-NPrs), were successfully developed via a robust and straightforward direct chemical reduction synthetic approach, producing desirable tiny and well-controlled Ag-NPrs. This procedure was accomplished by fabricating a mixture of di-sodium succinate hexa-hydrate (DSSH) and tri-sodium citrate di-hydrate (TSCD) as capping agents at optimal synthetic conditions and under an open-air condition, which proved to be an enormous challenge. Additionally, the Ag-NPrs were fully characterized by UV-vis spectra, X-ray diffraction (XRD), scanning electron microscope (SEM), and dynamic light scattering (DLS). Likewise, the formation stages from spherical silver nanoparticles (Ag-NPs) to triangular Ag-NPrs were also captured simultaneously via transmission electron microscope (TEM) and high-resolution transmission electron microscope (HR-TEM) images. More interestingly, an active thin silica-shell was efficiently applied on the Ag-NPrs outer-layer to increase their functionality. Furthermore, to confirm their biocompatibility, we also carried out cell viability assays for the Ag-NPs, Ag-NPrs, and Ag-NPrs@SiO_2_ with different concentrations at 62.5, 125, and 250 µg/mL after 12, 24, and 48 h of exposure time, respectively, on a regular African green monkey kidney cell line. The cell viability test results exemplified that the three silver nanostructures were toxic-free and suitable for further potential biological applications in the near future.

## 1. Introduction

Noble metal nanoparticles have received considerable attention over the past decade because of their exceptional chemical, optical, and electronic properties [1]. The previously mentioned assets have made them competent to be promoted by many remarkable applications in diverse research arrays, such as catalysis process [2], biological and chemical sensing [3,4], optics [5], and Surface-Enhanced Raman Spectroscopy (SERS) [6]. Since the fabrication of different shapes of metallic nanostructures has increased throughout the last few years, many routes have been swiftly developed to yield these magnificent two-dimensional (2D) structures, such as disks [7,8], rods [9], wires [10,11], and nanoprisms [12,13]. Lately, researchers have paid much attention to the synthesis and optical characterization of triangular silver nanoprisms (Ag-NPrs) or plates as they have excessive biological properties; likewise, Ag-NPrs can be efficient in bio-studies [14] as well. Attributable to their anisotropic shape, Ag-NPrs also have lavish plasmonic features in both visible and IR regions, and have shown significant SERS signals. Additionally, the relation between the lateral dimension and the thickness of the silver nanoprisms has an anisotropy property that favors a high turnability for their plasmonic band only when the lateral dimension is larger than the width [15,16], and their sharp tip morphology characteristics show potential in possible biomedical applications.

By focusing on the synthesis of Ag-NPrs, a vast number of procedures have been reported to fabricate distinct Ag-NPrs, including photo-induced aggregation [17], chemical reduction routes [18,19], and synthetic thermal routes [20]. The photo-induced aggregation procedures have been used to synthesize Ag-NPrs comprising the transformation of small nanoparticle seeds through photochemical synthesis processes [21,22]. These methods have generated high-quality Ag-NPrs during the synthesis; the reactions frequently occurred in dark rooms for an extended time of up to 2 months [23] and an authoritative light source was needed in large-scale synthesis [24]. 

Alternatively, the direct chemical reduction approach was considered the most acceptable technique due to its robust, cheap setup and applicable features for achieving large-scale assembly [25,26]. This route also comprised the gradual conversion of spherical silver nanoparticles (Ag-NPs) into triangular silver nanoprisms (Ag-NPrs). In 2005, Me´traux and Mirkin adopted the chemical reduction route to prepare Ag-NPrs using a mixture of AgNO_3_/NaBH_4_/polyvinyl pyrrolidone (PVP)/tri-sodium citrate (Na_3_CA)/H_2_O_2_ in aqueous solution at room temperature. However, their results revealed that the research group was incapable of obtaining small size and good homogeneous Ag-NPrs at large-scale [27]. 

Moreover, to enhance the stability and afford tunable solubility in various solvents, durable surface chemistry was cautiously developed to guard the Ag^0^ and Ag-NPrs against aggregation and different environmental damage [28]. Therefore, a silica shell coating can be extensively accomplished on the Ag-NPrs outer-layer. This silica shell (SiO_2_) is one of the most active di-electric layers [29,30], which has been widely investigated due to its unique chemical and biocompatible properties. In 2007, Xue et al. studied the surface modification of Ag-NPrs by immersing them into an ethanolic solution that contains 16-mercaptohexadecanoic acid (MHA) [31]. Lately, the biosafety and cytotoxicity assessment of silver nanocrystals has received prime research attention. Compared with other noble metals, silver is considered non-toxic and possesses efficient antimicrobial [32], antibacterial [33], and odor-fighting properties [34], which has inspired researchers to use these silver nanoparticles widely in countless consumer products, such as wound dressing [35], detergents [36], and antimicrobial coatings [37]. The most recent studies suggested that silver nanomaterials (Ag-NMs) were non-toxic as well, but some other studies showed them to be harmful to the environment [38]. In 2010, Wentong Lu et al. studied the cytotoxicity effect of Ag-NPs and Ag-NPrs and concluded that both Ag-NPs and Ag-NPrs were non-toxic to human skin cells [39].

In this study, we fabricated a suitable and direct chemical reduction synthetic approach via the combination of two capping agents, for instance, di-sodium succinate hexa-hydrate (DSSH) and tri-sodium citrate di-hydrate (TSCD), to synthesize well-dispersed Ag-NPrs, all the way from spherical-shaped silver nanoparticles to triangular-shaped silver nanocrystals, at room temperature within 5 min reaction time. To approve the Ag-NPrs synthesis for advance surface functionalization and bio-conjugation, an active ultra-thin silica shell layer was applied on the outer surface of the Ag-NPrs without notable red shifting being noticed. Meanwhile, transmission electron microscope (TEM), high-resolution transmission electron microscope (HR-TEM), X-ray diffraction (XRD), scanning electron microscope (SEM), and UV-spectra were conducted on the Ag-NPs and Ag-NPrs. Finally, the cytotoxicity of three different silver nanostructures, Ag-NPs, Ag-NPrs, and Ag@SiO_2_ NPrs, were successfully studied, which were tremendously valuable in bio-application studies.

## 2. Material and Methods.

Silver nitrate (AgNO_3_, 99%), polyvinyl-pyrrolidone (PVP, K-30) weight average molecular weight Mw ≈ 30, 000 g/mol, were purchased from Sigma-Aldrich Co., St. Louis, MO, USA. The di-sodium succinate hexa-hydrate (DSSH, 99%), tri-sodium citrate di-hydrate (TSCD, 99%), sodium borohydride (NaBH_4_, 96%), chloroform, methanol, ethanol, dimethylsulfoxide (DMSO), aqueous ammonia solution (28%), and hydrogen peroxide (H_2_O_2_, 30 wt %) were purchased from Sino pharm Chemical Reagent Co., Ltd, Shanghai, China. Sodium silicate solution (27 wt % SiO_2_) and N-octyltriethoxy silane (OTES, 98%) were purchased from Aladdin Industrial Co., Shanghai, China. The MTT 3-(4,5-dimethylthiazol-2-yl)-2,5-diphenyl tetrazolium bromide, was purchased from Sigma-Aldrich Co., St. Louis, MO, USA. The streptomycin, penicillin, trypsin, fetal bovine serum (FBS) and EDTA were purchased from GIBCO Invitrogen Co., Thermo Fisher Scientific, Foster, CA, USA. Dulbecco’s Modified Eagle Medium (DMEM) was purchased from Sino-American Biotechnology Co., China. An African green monkey kidney cell line (Vero cells), as a reference of normal cells, was purchased State Key Laboratory of Agricultural Microbiology, College of Veterinary Medicine, Huazhong Agricultural University, Wuhan, China. Cell culture 96-well plates were purchased from Corning Inc., New York, NY, USA. Ultra-pure water (Direct-Pure Water System, RephiLe Bioscience, Ltd., Beijing, China) with an 18.2 MΩ·cm resistivity was used through all experiments. All chemicals and reagents were used as received without further purification.

### 2.1. Synthesis of Triangular Silver Nanoprisms (Ag-NPrs)

A new systematic synthesis approach was fabricated to achieve the well-formed shape of the Ag-NPrs. In brief, the total volume of the reaction solution was fixed at 10 mL. Typically, a 9.99 mL aqueous solution combining silver nitrate (0.01 M, 0.1 mL), tri-sodium citrate di-hydrate (TSCD, 75 mM, 0.125 mL), di-sodium succinate hexa-hydrate (DSSH, 0.1 M, 0.025 mL) H_2_O_2_ (30 wt %, 0.0245 mL), and poly(vinylpyrrolidone) (PVP, weight average molecular weight Mw ≈ 30,000 g/mol, 17.5 mM, 0.045 mL) was vigorously stirred at room temperature in open-air conditions. After 2 min, sodium borohydride (NaBH_4_, 100 mM, 0.1 mL) was rapidly injected into this mixture to initiate the reduction, immediately leading to a light-yellow solution. Within 2–3 min, the color changed from light yellow to deep yellow and gradually to blue, due to the Ag-NPs aggregation and the formation of Ag-NPrs within 5 min reaction time. Likewise, the solid state of the silver nanoprisms can be obtained after the final formation of silver nanoprisms by centrifuging the aqueous solution of the blue Ag-NPrs for 5 min at 10,000 rpm, then discarding the supernatant and freeze drying the precipitant for further procedure.

### 2.2. Characterization of Triangular Silver Nanoprisms (Ag-NPrs)

The morphological shape and formation mechanism of Ag-nanostructure materials were characterized by using Transmission Electron Microscope (TEM, JEM-2100, Akishima, Tokyo, Japan) and High-resolution Transmission Electron Microscope (HR-TEM, FEI Talos F200C at 200 kV, Tokyo, Japan) and scanning electron microscopy (SEM, JSM-6700F, Akishima, Tokyo, Japan). The XRD analysis was performed with a Bruker D8 Advance X-ray diffractometer equipped with a Cu Ka radiation source (Karlsruhe, Germany). The measurements of optical properties were conducted by using a PerkinElmer UV/VIS Spectrometer Lambda25, manufactured by PerkinElmer, Ayer Rajah Crescent, Singapore Pte Ltd., and UV-2450 UV-vis Shimadzu spectrophotometer, Kyoto, Japan. Meanwhile, the hydrodynamic size and zeta potential were also measured by dynamic light scatting (DLS) using a Nano-ZS ZEN3600 Malvern Instruments, Worcestershire, UK. Also, the measurements of the cell viability were carried out by using Enzyme-Linked Immunosorbent Assay (ELISA, Thermo Fisher Scientific, Waltham, MA, USA) at 570 nm wavelength.

### 2.3. Silver Nanoprisms Silica Coating (Ag-NPrs@SiO_2_)

The silica coating step was achieved by salinization with lipophilic silane in a stepwise method followed by sodium silicate deposition in water. Momentarily, 500 μL (1.4 × 10^−5^ mol/L) of Ag-NPrs was precipitated using 1:1 methanol/sample mixture. This phase was repeated three times and lastly washed with dd H_2_O. In all three steps, the supernatant was discarded, and the residue was moderately evaporated via air flow. The precipitate was at that point dissolved into 20 μL of OTES using the mini-sonicator (L10-300A, Shanghai, China) for 3 seconds to enable the solubilization process. Then, 20 mL of water was introduced along with 30 μL of NH_3_·H_2_O, after which the combination was stirred for 1h. The resulting solution was passed through two different types of filters, a 0.22 μm pore size filter to eliminate any standing cloudiness and a 0.42 μm pore size filter to eradicate any remaining hollow silica. Afterward, a total volume of 0.8 mL of a 0.54 wt % sodium silicate aqueous solution was injected into the solution with 6 h continuous stirring. Finally, circles of ultra-filtration through a 30 kDa MWCO Amicon filter were conducted to purify the crude solution of any excessive silica beads for further applications.

### 2.4. Cell Viability Assay

In this section, Vero cells were cultured in 96-well plates and incubated for 12 h before experiments until the cell growth reached approximately 85% confluence. Different silver nanostructures (Ag-NPs, Ag-NPrs, and Ag-NPrs@SiO_2_) were dispersed into Dulbecco’s Modified Eagle’s Medium (DMEM) with different concentrations of 62.5, 125, or 250 µg/mL. Vero cells then were incubated at 37 °C with 5% CO_2_ atmosphere for three different time intervals at 12, 24, or 48 h, respectively. The main purpose of dispersing different silver nanostructures in DMEM is to test their stability, as this media represents the growing environment for the cells under investigation and enables study of the cell viability, to clarify the biocompatibility of the silver nanostructures for future studies. After the incubation period, all wells were washed with PBS to remove excess silver nanostructures and placed in a fresh solution of 200 μL of PBS before the next experiments. Afterward, the cell viability was determined by using MTT assay. Momentarily, a 20 μL MTT stock solution (0.005 µg/mL) was added to each well, and cells were then incubated for an extra 4 h at 37 °C. Lastly, the supernatant was discarded, and a 150 μL/well of DMSO was added to dissolve the formazan, the artificial chromogenic products of the reduction of tetrazolium salts by dehydrogenases and reductases, and the plates were gently shaken for an additional 10 min. Consequently, the absorbance of the purple formazan was recorded via ELISA at 570 nm.

## 3. Results and Discussion

### 3.1. Preparation of Triangular Silver Nanoprisms (Ag-NPrs)

**Figure 1:**
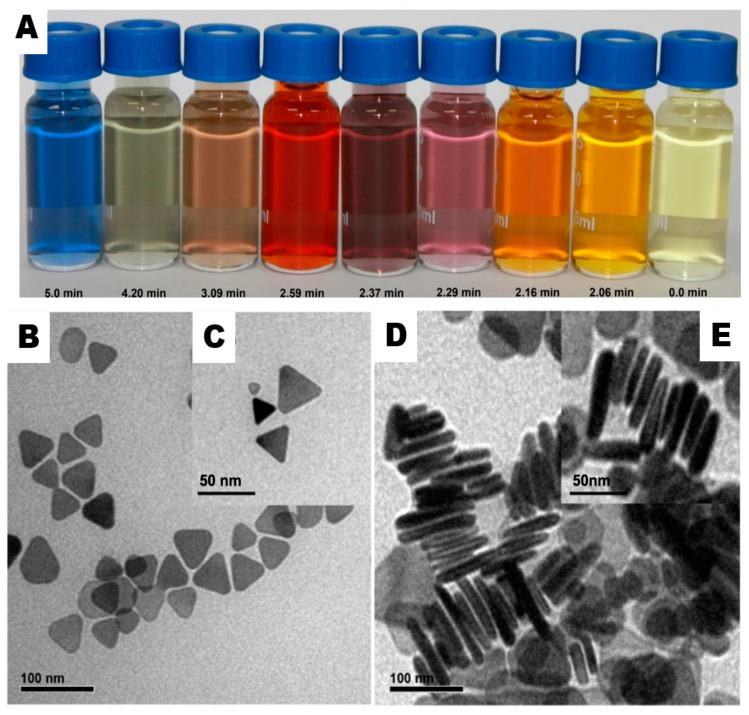
(**A**) Digital photo of Ag-NPrs prepared in the presence of the mixture of (0.025 mL) tri-sodium citrate di-hydrate (TSCD) and (0.125 mL) di-sodium succinate hexa-hydrate (DSSH). Total bottle volume (1.5 mL), (**B**) transmission electron microscope (TEM) of silver nanoprisms, (**C**) high-resolution transmission electron microscope (HR-TEM) of Ag-NPrs (inset), and (**D**) TEM of Ag-NPrs were standing vertically face-to-face. (**E**) HR-TEM of vertically standing Ag-NPrs (scale bar in B–D images are 50 nm).

To synthesize well-edged Ag-NPrs, a new robust chemical reduction route was developed to overcome the challenges in previous studies, for instance aggregation, etching, colloidal stability, and red shifting, by combining two capping agents, for example, di-sodium succinate hexa-hydrate (DSSH) and tri-sodium citrate di-hydrate (TSCD), with a ratio of (1:5) at room temperature and open-air conditions. Together with other solution components and under vigorous magnetic stirring, a solution of silver nitrate (AgNO_3_), polyvinyl-pyrrolidone (PVP), and hydrogen peroxide (H_2_O_2_) were mixed first for two minutes. Sodium borohydride (NaBH_4_) has been rapidly injected into the previous mixture to initiate the reduction mechanism. Within exactly 5 min reaction time, a strong blue color was achieved successfully. As shown clearly in the digital photo of Figure 1A, the solution showed a clear light-yellow color first, but gradually transformed into deep yellow, violet, purple, red, brown, green, and finally blue within 5 min reaction time. These distinctive colors of silver nanostructure are because of a unique phenomenon well-known as plasmon absorbance, where incident light generates fluctuations in conveyance electrons on the surface of the nanoparticles and electromagnetic radiation is captivated. This clarifies the robust synthesis of Ag-NPrs in relevant time in comparison with other synthetic approaches that may take over a day or so, and overcomes the aggregation and redshift defects [23,40,41,42]. 

### 3.2. Characterization of Ag-NPrs

**Figure 2:**
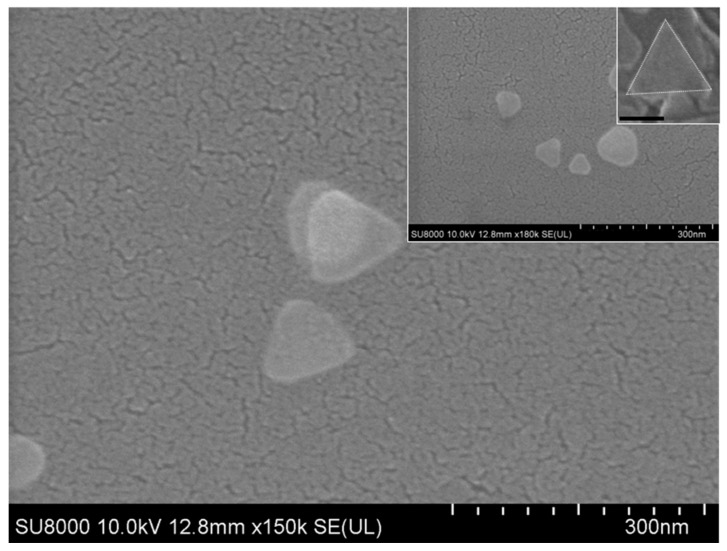
SEM pattern of the as-prepared silver nanoprism Ag-NPrs (the inset scale bar 300 nm).

Herein, the imperfection of engineering homogenous and tiny Ag-NPrs at large-scale [27] was taken into consideration, and we were able to achieve size-controlled Ag-NPrs to the best of our knowledge. As revealed in Figure 1B,C, the transmission electron microscopy (TEM) and high-resolution transmission electron microscopy (HR-TEM) images showed that the as-prepared Ag-NPrs obtained an average size from ~3 to 35 nm. More interestingly, Figure 1D, and HR-TEM in the inset (Figure 1E), showed face-to-face Ag-NPrs standing vertically upon their edges, as the vertical orientation was acquired when the surface pressures were high [31]. Conveniently, we could estimate their thickness, which wavered from 2–5 nm in diameter. Similarly, the SEM images illustrated in Figure 2 and the inset confirmed the Ag-nanoprism’s shape.

**Figure 3:**
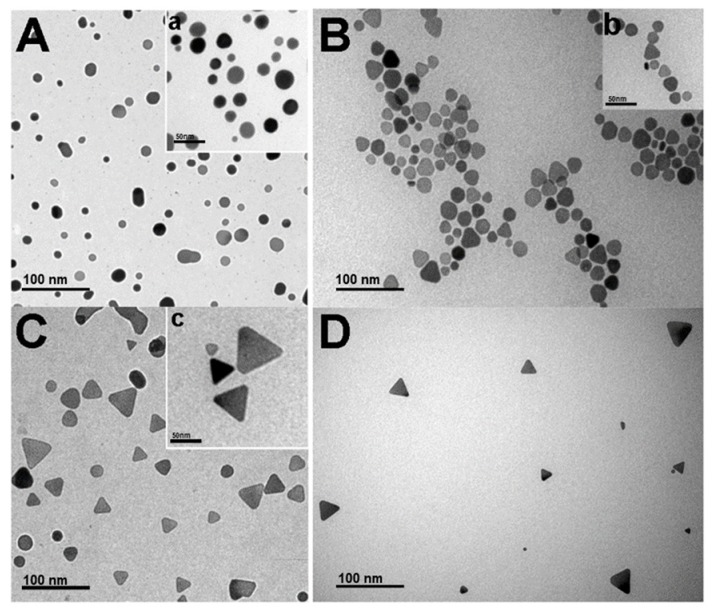
TEM and HR-TEM of Ag Nanoparticles (**A**,**a**), intermediate phase (**B**,**b**), Ag Nanoprisms (**C**,**c**), and (**D**) TEM of Ag-NPrs after washing (the inset a,b,c scale bar is 50 nm).

**Figure 4:**
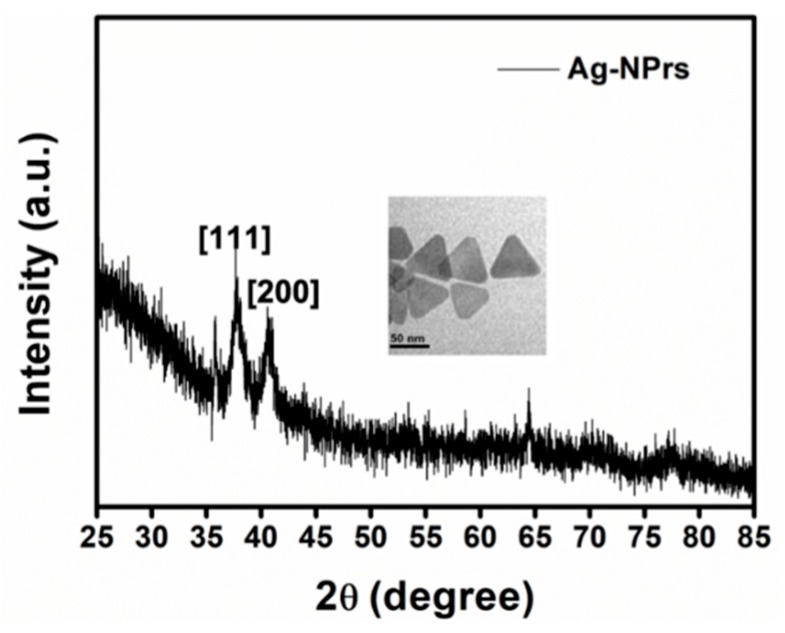
X-ray diffraction (XRD) pattern of triangular silver nanoprisms (Ag-NPrs); inset is the Ag-NPrs TEM image.

Likewise, we came across the formation mechanism of the triangular nanoprisms by achieving an intermediate formation stage for the silver nanoprisms (Ag-NPs, the intermediate phase, Ag-NPrs, and an HR-TEM image of Ag-NPrs), as presented in Figure 3. This suggests that within 5 min reaction time the formation of the Ag-NPrs can be carried by the chemical reduction route, which to our knowledge, is the first to be reported. Moreover, the edged Ag-NPrs crystalline evidence was attained via a monolayer XRD diffraction measurement. As shown in Figure 4, two characteristic intensive peaks at 2θ degree at 38.5° and 41.5° corresponded to the crystallize reflections of Ag^0^, in the face-centered cubic (fcc) structure with lattice planes (111) and (200) of silver nanoprisms (JCPDS File no. 4-0862), respectively. This is an effervescent indicator to the formation of pure, composed crystalline silver nanoprisms. Additionally, the first peaks confirmed that the silver nanoparticles were not a sphere anymore but positioned along the (111) plane to form the structure of the nanoprisms, agreeing well with our TEM and HR-TEM results. Conveniently the narrower the XRD peaks, the larger the size of the nanoprisms, which was consistent with the Scherrer equation *D* = *k*λ/βcosα as well [43].

**Figure 5:**
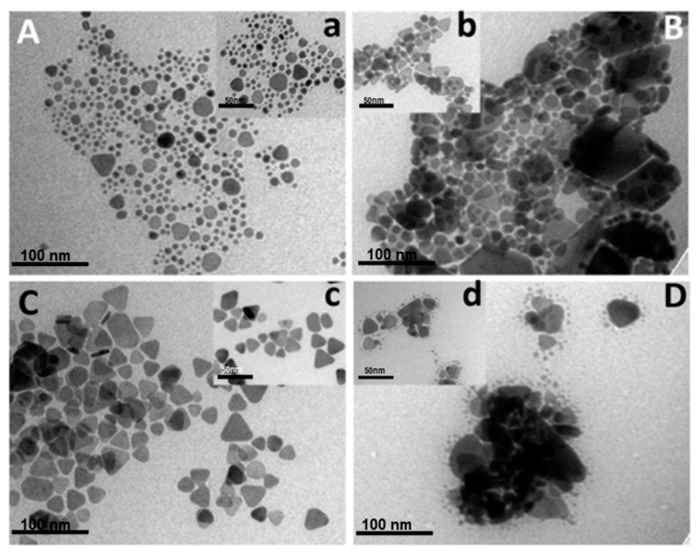
TEM images (**A–D**, scale bar 100 nm) and inset (**a**–**d**, scale bar 50 nm) show the HR-TEM images of Ag-NPrs obtained in the four concentrations of tri-sodium citrate di-hydrate (TSCD) and di-sodium succinate hexa-hydrate (DSSH) ratio.

From all ratios that have been utilized in Table 1, TEM and the inset HR-TEM images in Figure 5C,c, respectively confirmed that No.3, which use a ratio of 1:5 for DSSH and TSCD, was the most exceptional ratio to acquire clear, morphological, well-defined Ag-NPrs. As evident from the synthesis ratio in Table 1, the TSCD ratio was 5-fold higher than the DSSH ratio, which has a tremendous influence on serving as a bi-functional, shape-directing, and stabilizing reagent through the reaction synthesis process, achieving a vibrant morphological nanoprisms shape.

### 3.3. UV-Vis Measurement of the Ag-NPrs Formation

**Figure 6:**
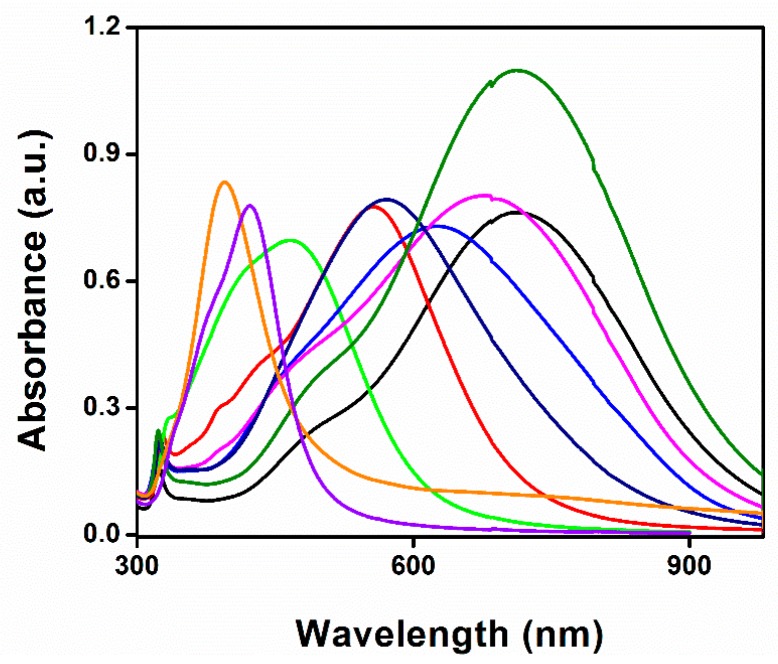
UV-vis spectra of Ag-NPrs formation with an average time interval of 21.75 s.

**Figure 7:**
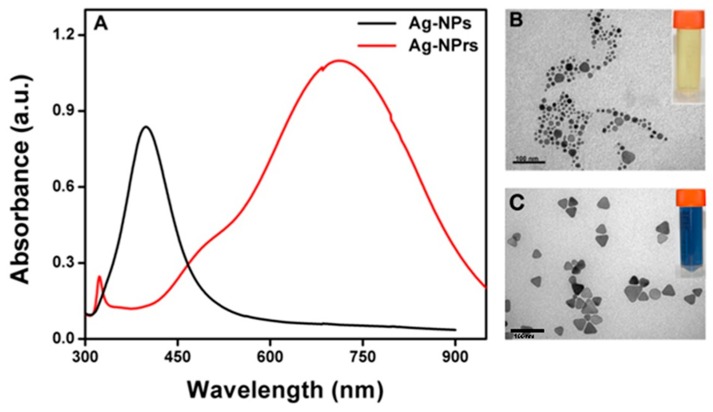
UV-vis spectra of Ag-NPs and Ag-NPrs (**A**) and TEM images are showing that both Ag-NPs with spherical shapes can be converted to Ag-NPrs at room temperature and open-air conditions (**B**,**C**).

A UV-Vis spectrophotometer was used to record various spectral deviations in the optical properties of the Ag-NPrs colloidal solution. As observed in Figure 6, a very robust absorption band in the short UV wavelength range < 400 nm was distinguished. In the next 1–2 min reaction time, the light yellow solution swiftly turned into deep yellow within several seconds, signifying the formation of the Ag-NPs. The strength of the individual peak at 400 nm was amplified rapidly when Ag-NPs formation started. The smaller silver nanospheres with light yellow color primarily absorbed light and had plasmon resonance peaks near 400 nm, while larger nanoprisms with blue color exhibited increased scattering and had peaks that broaden and shift towards longer wavelengths at 750 nm. Through exactly controlling the silver nanoprisms’ thickness and diameter, the plasmon resonance can be adjusted to peak at exact wavelengths [44,45]. A few seconds later, the UV-Vis spectra changed, as illustrated in Figure 6, indicating a gradual variation in the solution color from deep yellow to violet, purple, red, brown, green, and finally, blue, as presented in the digital image in Figure 1A. The transformation of spherical silver nanoparticles during the reaction clarified the decrease of their spectral peak. Nevertheless, the new peak at ~450 nm appeared and regularly increased to a longer wavelength, while at the same time the formation and growth of Ag-NPrs was acquired; within 5 min reaction time, the Ag-NPrs absorption peak emblem increased to its maximum, situated at the 750 nm wavelength. The UV-Vis measurements of the colloidal nanoprisms reflected their anisotropic shape. Conclusively, the difference between the UV-Vis spectra of Ag-NPs and Ag-NPrs can be summarized in two main characteristic peaks located at 400 nm and 750 nm, respectively, as shown in Figure 7A. Additionally, the TEM images of the spherical Ag-NPs and triangular Ag-NPrs and their clear inset digital photos were presented in Figure 7B,C.

### 3.4. Size-Dispersal of Ag-NPrs

**Figure 8:**
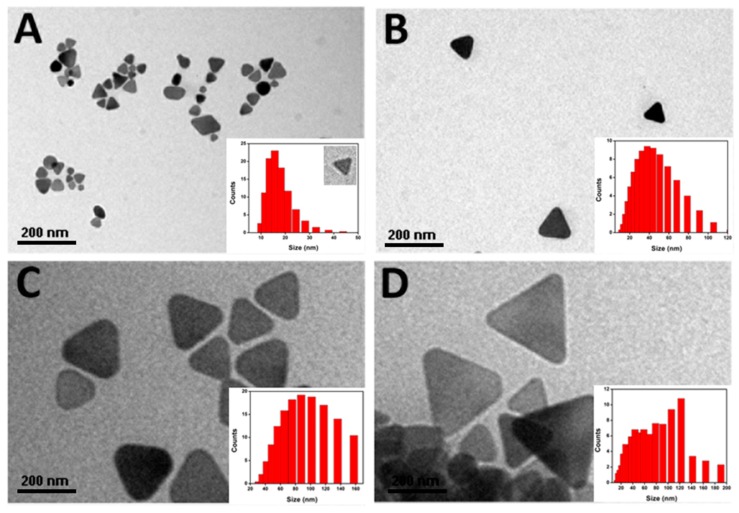
Representative TEM images of Ag-NPrs (**A**) ~11 nm, (**B**) ~44 nm, (**C**) ~90 nm, and (**D**) ~120 nm, with the corresponding size distribution of the Ag-NPrs inset.

A zeta sizer was used to measure the synthesized Ag-NPrs size in the hydrophilic phase, which represented the core synthesis phase. The steps for preparing the hydrophilic solution of Ag NPrs was by dissolving 1mg from the solid-state Ag NPrs in 1 mL distilled water, followed by 2 min sonication, then 10 µL was placed over copper grids and left to dry for TEM measurements. A perfect shape for Ag-NPrs with an average tunable-size from ~3–35 nm was successfully achieved, which is considered entirely different from other reported results [46,47]. Meanwhile, Figure 8 represents TEM images and the corresponding size distribution of the Ag-NPrs under investigation is inset. The average zeta size range of the Ag-NPrs ranged from ~11 to 44 nm, as presented in Figure 8A,B. The size of the Ag-NPrs appears to be larger in the hydrophilic media as compared to their dry size from TEM observation, which is perhaps instigated from the existence of an electrostatic bilayer in the Ag-NPrs hydrophilic solution [48]. Also, the lateral dimensions of Ag-NPrs can be controlled progressively by adjusting the stirring time at room temperature as shown in Figure 8.

### 3.5. Silica Shell Formation and UV–Vis Spectra of Ag-NPrs, before and after Coating

**Figure 9:**
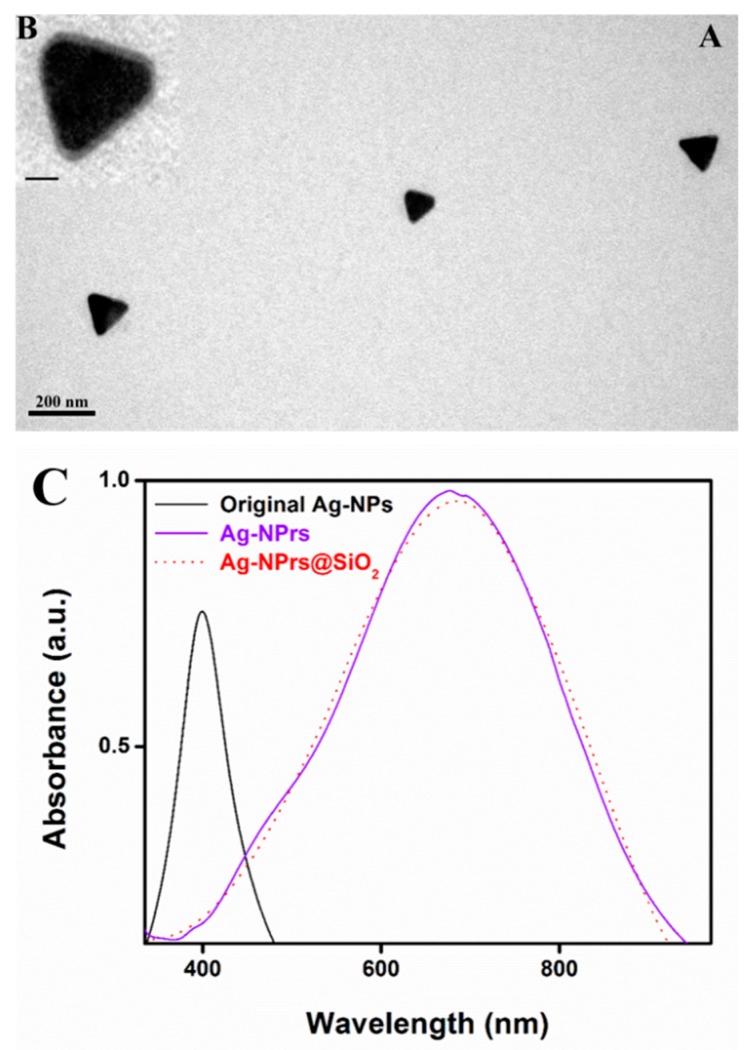
(**A**) TEM image of silica coated Ag-NPrs with a silica shell from 3–6 nm, (**B**) inset with the HR-TEM image of silica coated Ag-NPrs (inset scale bar 50 nm). (**C**) The UV-vis spectra of the Ag-NPs, Ag-NPrs, and Ag-NPrs@SiO_2_ at two different wavelengths (400 and 750 nm, respectively).

A silica shell was applied on the outer surface to protect the Ag-NPrs from aggregation, agglomeration, etching, and environmental damage [49]. The growth of the silica shell was achieved in an ammonium solution using alkoxysilane chemistry with the assistance of N-octyltrimethoxysilane (OTES) [48,50]. A well-formed and ultra-thin silica shell layer was produced, with width ranging from 3 to 6 nm. The Ag-NPrs@SiO_2_ was successfully achieved, as illustrated in the TEM image in Figure 9A and the inset HR-TEM images (Figure 9B). Also, by increasing the stirring time, a thicker silica shell was obtained. Likewise, a UV-Vis spectroscopy was used to measure the consistent changes in the optical properties of the naked and silica-coated Ag-NPrs. An effective direct silica coating was attempted on the outer surface of the Ag-NPrs, without any significant red-shift noticed between the original spectra of the Ag-NPrs and the silica-coated Ag-NPrs@SiO_2_, as presented in Figure 9C, which was attributed to the addition of PVP into the initial synthesis process. In this manner, the direct silica coating of the Ag-NPrs using OTES presented an almost identical spectrum with the naked Ag-NPrs, which allowed us to overcome the red-shift that is acquired by using a modifying surfactant, for instance, MHA [31]. 

### 3.6. Cytotoxicity of Ag-NPrs before and after Silica Coating

**Figure 10:**
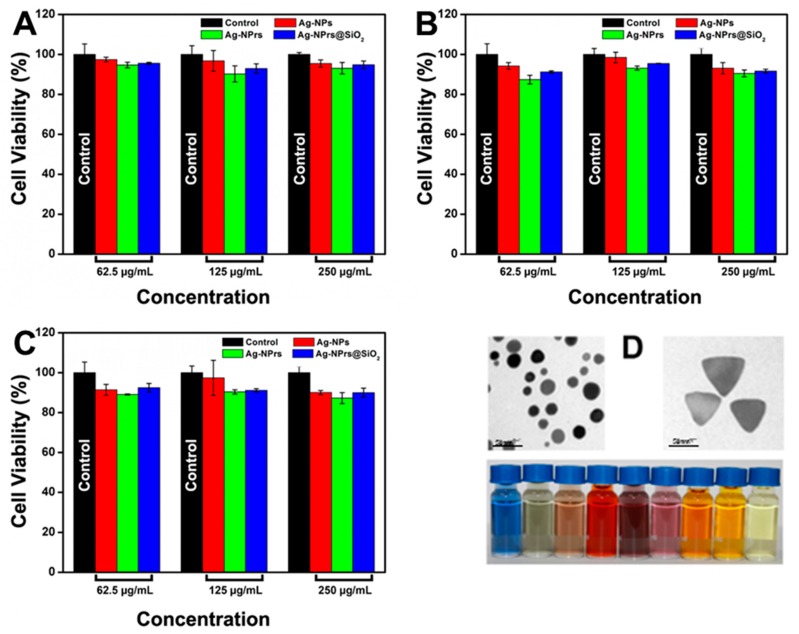
(**A**) The cell viability of Ag-NPs, Ag-NPrs, and Ag-NPrs@SiO_2_ with 62.5 µg/mL, 125mL, and 250 µg/mL concentrations after 12 h exposure time. (**B**) The cell viability of Ag-NPs, Ag-NPrs, and Ag-NPrs@SiO_2_ at the concentrations of 62.5, 125, and 250 µg/mL after 24 h exposure time. (**C**) The cell viability of Ag-NPs, Ag-NPrs, and Ag-NPrs@SiO_2_ at concentrations of 62.5, 125, and 250 µg/mL after 48 h exposure time. (**D**) Overall Ag-NPrs formation TEM image and digital photo used for the cell viability assay. Cell viability assays were triplet repeats.

An MTT assay was carried out to evaluate the cytotoxicity effects of the newly developed type of Ag nanostructures on Vero cells, an African green monkey normal kidney cell line. Briefly, three Ag nanostructures (Ag-NPs, Ag-NPrs, and Ag-NPrs@SiO_2_) with 62.5, 125, or 250 µg/mL concentrations were added simultaneously to the cultivated cells in 96-well plates and incubated for 12, 24, or 48 h, correspondingly. Meanwhile, all control groups contained Vero cells with culture broth without any nanoparticle treatment. As presented in Figure 10A,B, there was nearly no significant cytotoxicity between the control group and the treated cells with the previously mentioned concentrations of Ag-NPs, Ag-NPrs, and Ag-NPrs@SiO_2_ in hydrophilic form after exposure for 12 and 24 h. Even after extending the exposure time to 48 h, no major diversity in the cell viability ratio was detected, as demonstrated in Figure 10C. This indicates that various concentrations of Ag-NPs, Ag-NPrs, and Ag-NPrs@SiO_2_ in aqueous forms were not innately toxic to normal cells, even after using a 2.5-fold higher concentration than the previous report [37], and were qualified for future biological labeling. Figure 10D also represented the spherical and the triangular nanostructure prisms that were applied in the viability assay. More interestingly, from the cytotoxicity test results, the cell viability of the Ag-NPrs@SiO_2_ was higher than the cell viability of the naked Ag-NPrs, as the silica coating enhanced the properties of Ag-NPrs and reduced the release of the silver ions into the medium, while preserving their colloidal stability and environmental damage as well. The cytotoxicity evaluation results outstandingly demonstrated that the obtained Ag-NPs, Ag-NPrs, and Ag-NPrs@SiO_2_ were biocompatible and applicable for potential bio-applications.

## 4. Conclusions

In this work, we reveal that the direct chemical reduction route can be conducted at room temperature and open-air conditions with the combination of two capping agents, DSSH and TSCD, at a 1:5 ratio, to fabricate small-sized and controllable Ag-NPrs, ranging from ~3 to 35 nm. Also, the formation stages from spherical silver nanoparticles to silver nanoprisms passing by the intermediate phase were captured by TEM and HR-TEM. More interesting, a uniform ultra-thin silica shell was additionally applied around the Ag-NPrs, with width ranging from 3 nm to 6 nm, for the first time. Furthermore, the cell viability for Ag-NPs, Ag-NPrs, and Ag-NPrs@SiO_2_ with three different concentrations of 62.5, 125, and 250 µg/mL after various exposure times of 12, 24, and 48 h was studied. Moreover, these studies provided a durable clue that the silica coating enhanced the stability and the biocompatibility of the Ag-NPrs, and the pre-synthesized Ag-NPrs and Ag-NPrs@SiO_2_ were non-toxic and could be used in approaching biocompatible applications.

## Figures and Tables

**Table 1:** Reaction conditions for the Ag-NPrs synthesis in a total volume of 10 mL aqueous solution.

No.	H_2_O	PVP (17.5 mM)	AgNO_3_ (0.01 M)	DSSH (0.1M)	TSCD (75 mM)	H_2_O_2_	NaBH_4_ (100 mM)
1	9.68 mL	0.045 mL	0.1 mL	0.05 mL	0.1 mL	0.0245 mL	0.1 mL
2	9.68 mL	0.045 mL	0.1 mL	0.1 mL	0.05 mL	0.0245 mL	0.1 mL
3	9.68 mL	0.045 mL	0.1 mL	0.025 mL	0.125 mL	0.0245 mL	0.1 mL
4	9.68 mL	0.045 mL	0.1 mL	0.125 mL	0.025 mL	0.0245 mL	0.1 mL
